# Supplementary material for: Multi-omic analyses reveal the unique properties of chia (Salvia hispanica) seed metabolism
Source: Commun Biol. 2023 Aug 7;6:820. doi: 10.1038/s42003-023-05192-4 (PMC10406817; doi:10.1038/s42003-023-05192-4)
Supplement: Supplementary file 2 — Supplementary Material [file 42003_2023_5192_MOESM2_ESM.pdf]

Multi-omic analyses reveal the unique properties of chia (*Salvia hispanica*) seed metabolism

Gerardo Alejo-Jacuinde, Héctor-Rogelio Nájera-González, Ricardo A. Chávez Montes, Cristian D. Gutierrez Reyes, Alfonso Carlos Barragán-Rosillo, Benjamin Perez Sanchez, Yehia S. Mechref, Damar López-Arredondo, Lenin Yong-Villalobos, Luis Herrera-Estrella

Supplementary Figures S1 to S16
Supplementary Tables S1 to S14

Supplementary Figure S1. Plant material used in this study. **a**) Adult *Salvia hispanica* plant, **b**) close up raceme and flowers, **c**) leaves, and **d**) dry seeds.

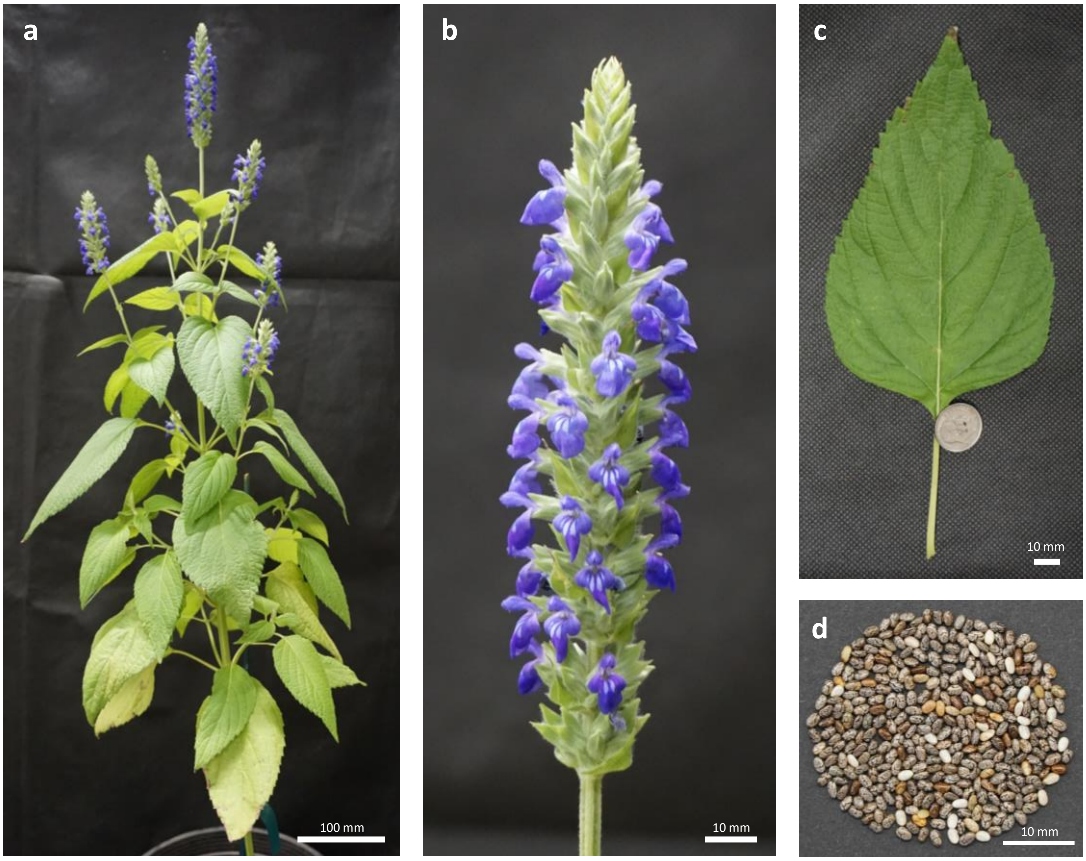

Supplementary Figure S2. Genome size estimation based on *k*-mer frequencies. Genome size was estimated by k-mer analysis (21-mer) using findGSE tool. This analysis determined a *Salvia hispanica* genome of 358.323 Mb.

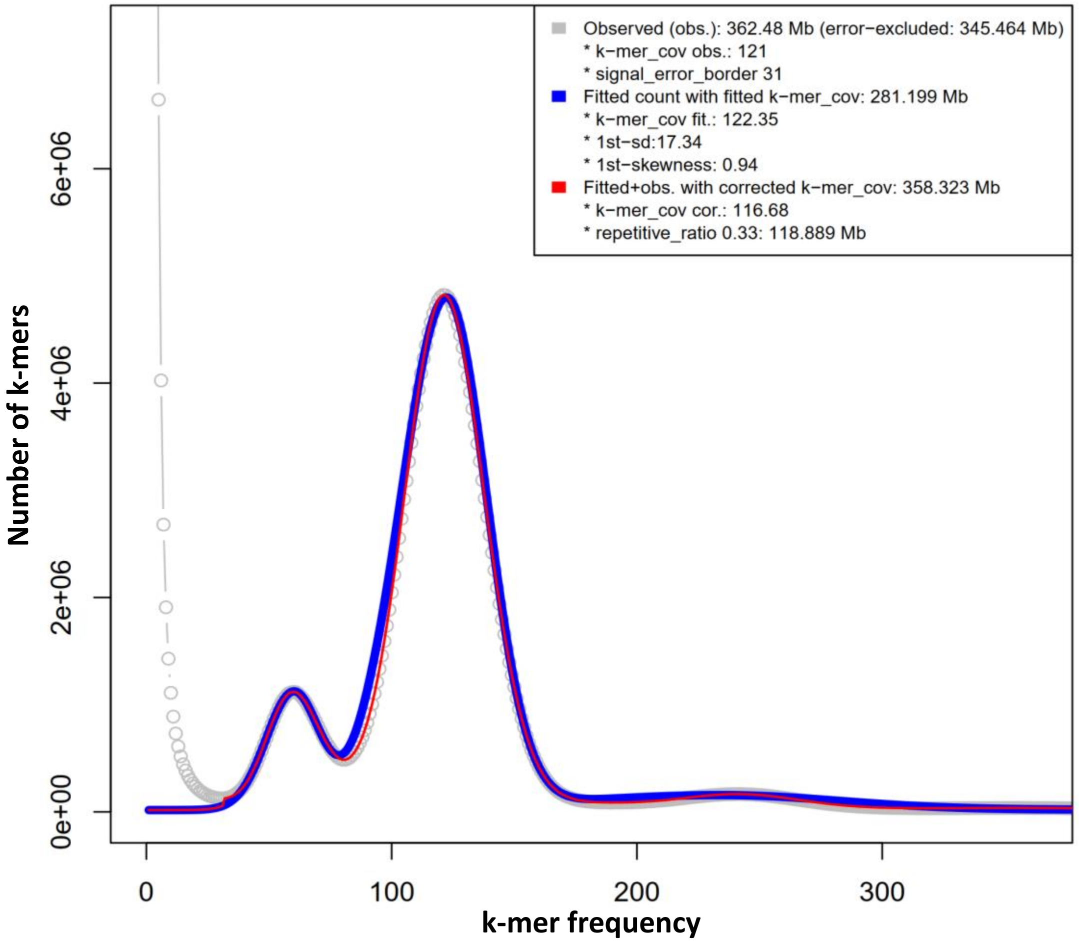

Supplementary Figure S3. Contig scaffolding using Hi-C data. Output of the Hi-C data analysis (Hi-C interaction map) visualized in Juicebox (v1.11.08). Pseudochromosomes (blue) were reconstructed ordering and joining contigs (green). The gray arrow indicates an interaction hotspot between chromosome chr1 and chr2.

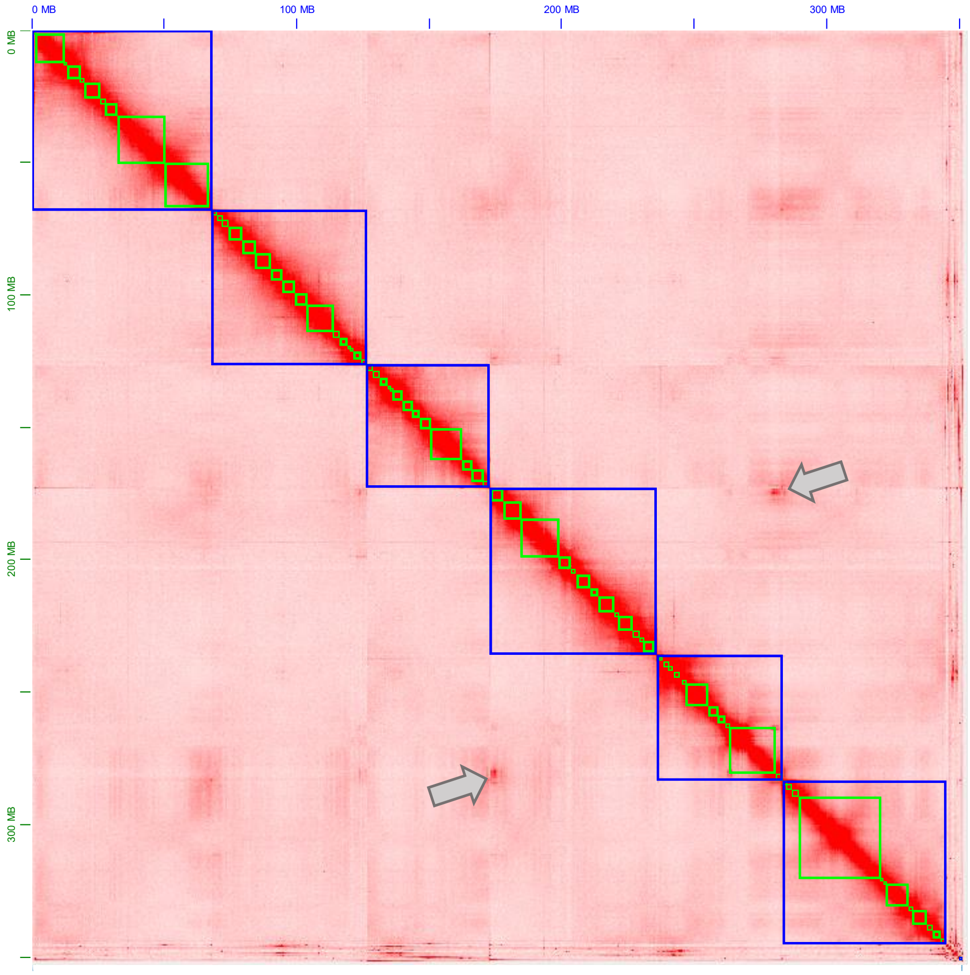

Supplementary Figure S4. Telomeres in *Salvia hispanica* genome assembly. Chromosomes that exhibit telomeric repeats are depicted. The number of repeats and their most common repeat per chromosome are indicated.

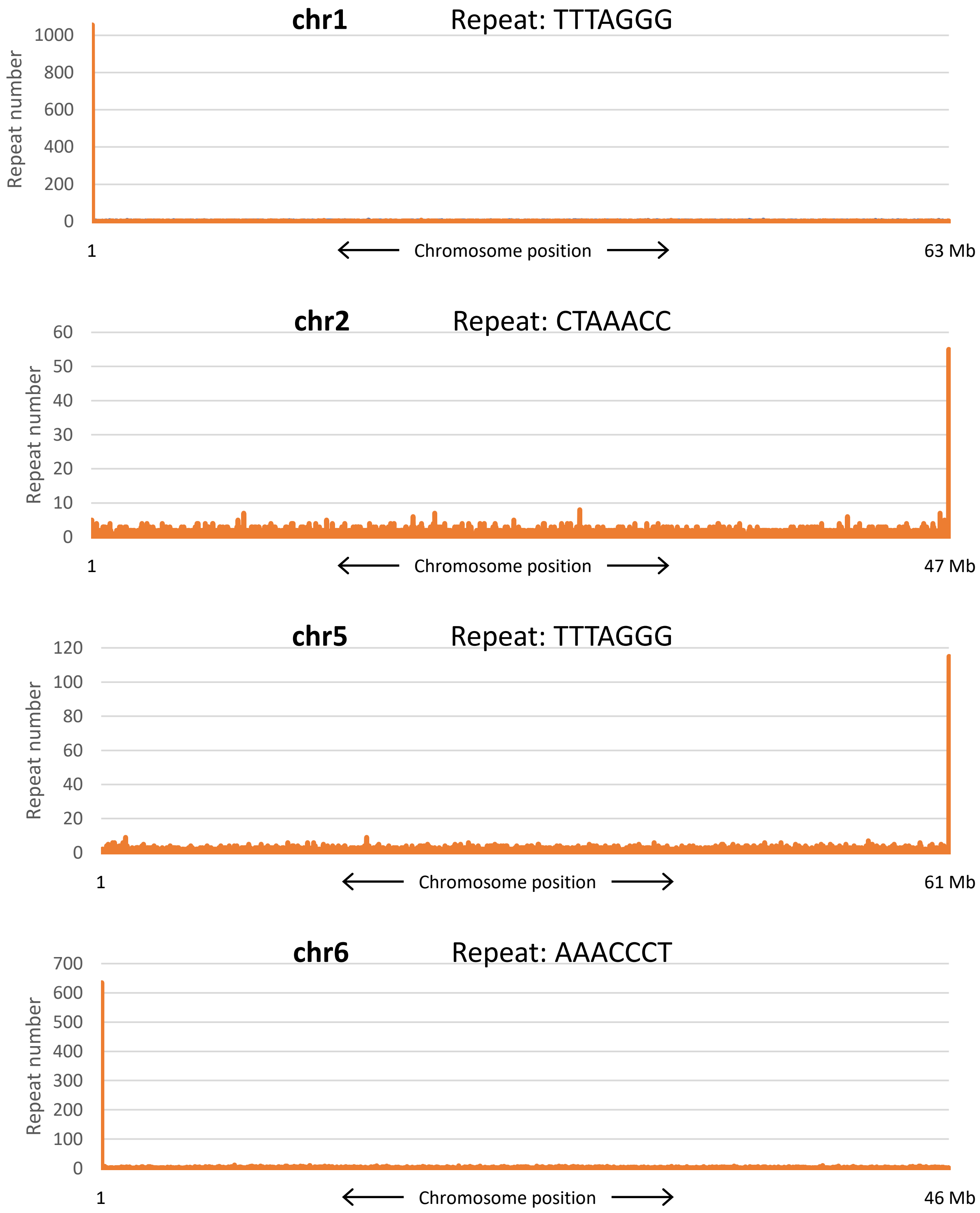

Supplementary Figure S5. Genome alignment between publicly available *Salvia hispanica* genome assemblies. Chia chromosomes were aligned using minimap2 and visualized with D-GENIES (https://dgenies.toulouse.inra.fr/). The genome assembly generated in this study was aligned to: **a**) the assembly reported by Wang *et al.*, (2022) and **b**) the assembly reported by Li *et al.*, (2023). The red, dashed circle highlights a 4.6 Mb fragment that was located in a different chromosome in the assembly reported by Wang *et al.*, (2022).

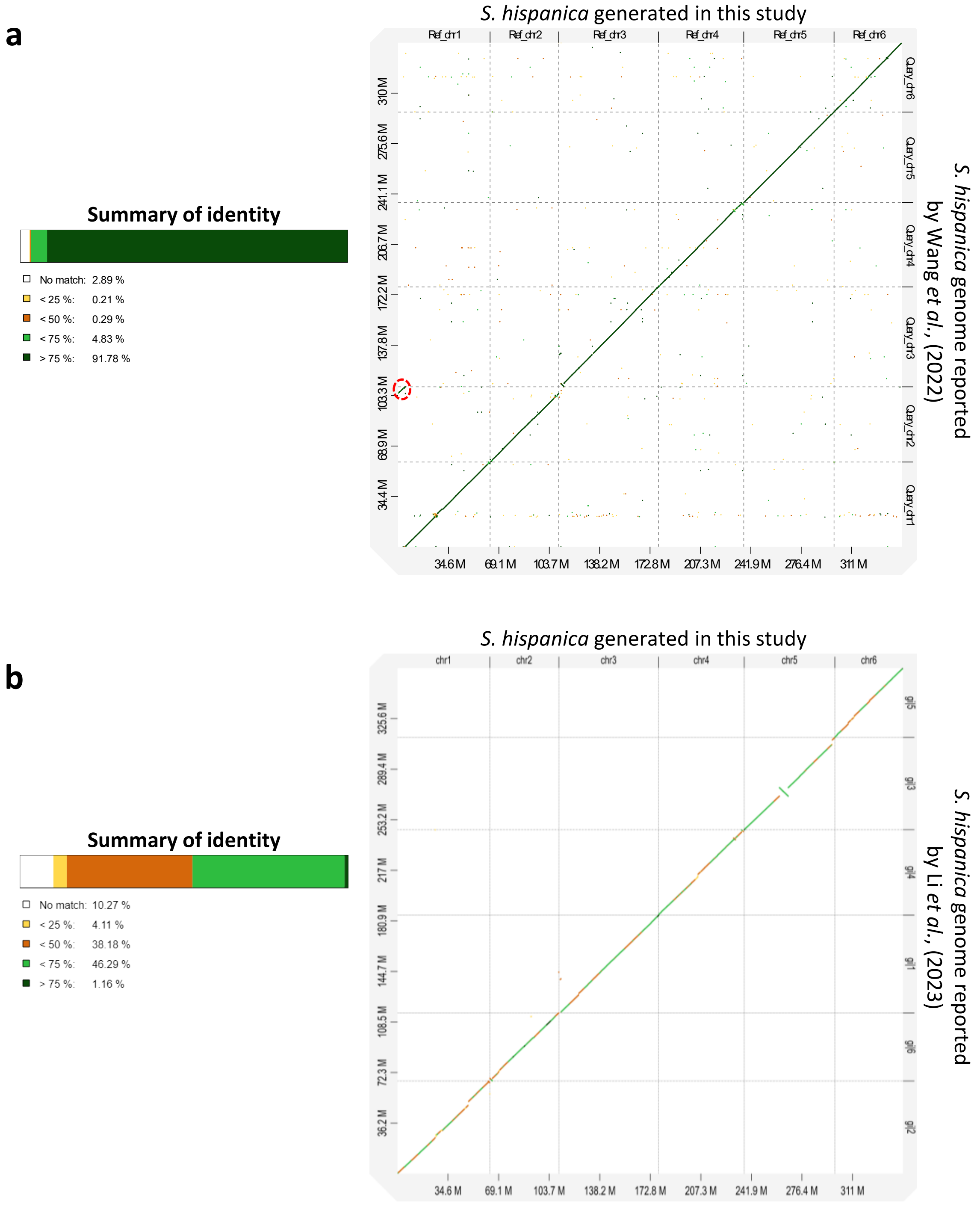

Supplementary Figure S6. Hi-C analysis to resolve the chromosome position of the 4.6 Mb fragment. **a**) Contact heatmap using Hi-C data generated in this study. **b**) Contact heatmap using data generated by Wang *et al*., (2022). The number of paired alignments between 4.6 Mb fragment with chr1 and chr2 is indicated.

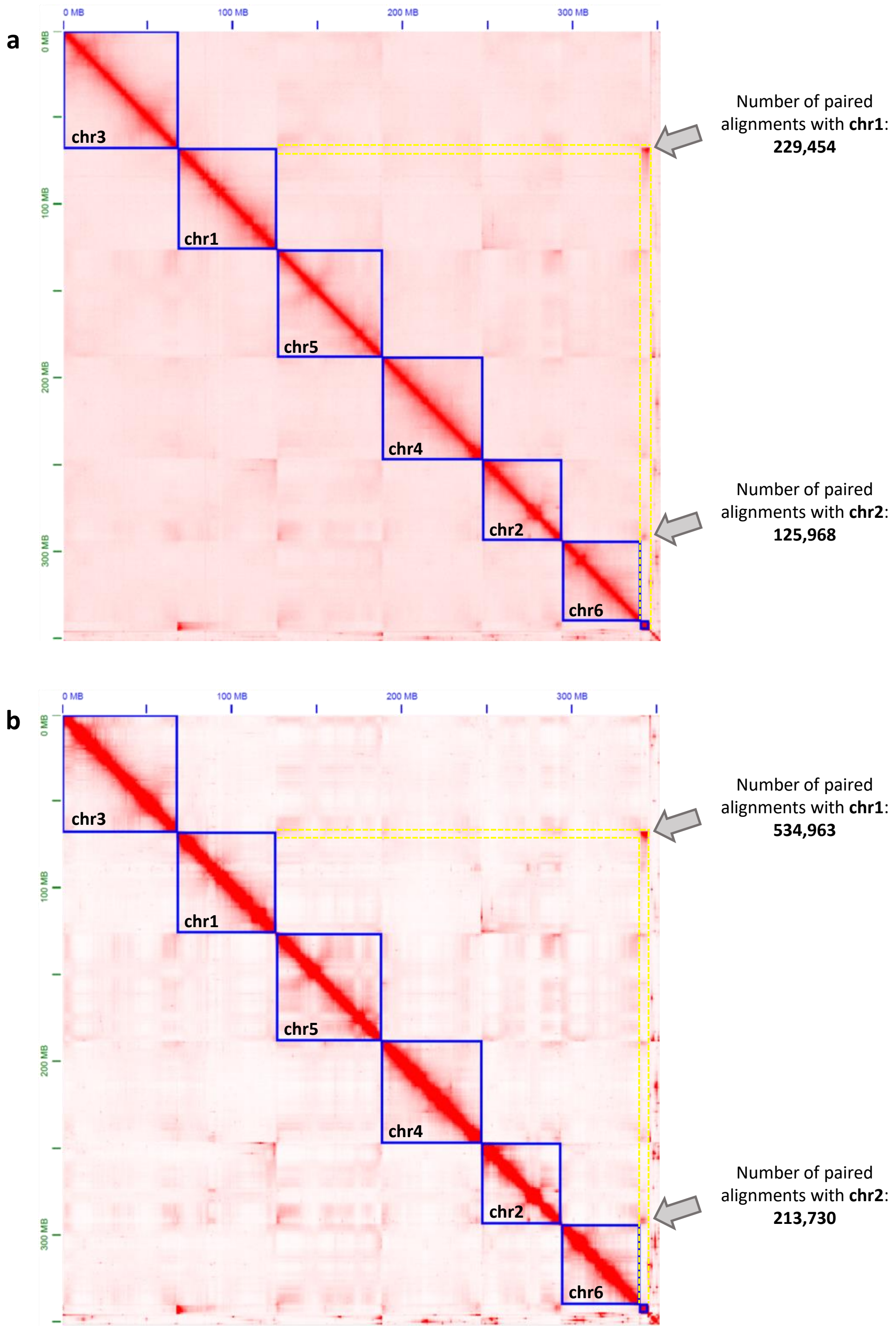

Supplementary Figure S7. Genome annotation completeness evaluated by BUSCO in publicly available *Salvia hispanica* genomes. Gene models predicted in this study (Mexican variety) and the previously reported chia genome [Australian variety reported by Wang *et al.*, (2022)] were evaluated using BUSCO with the embryophyta dataset.

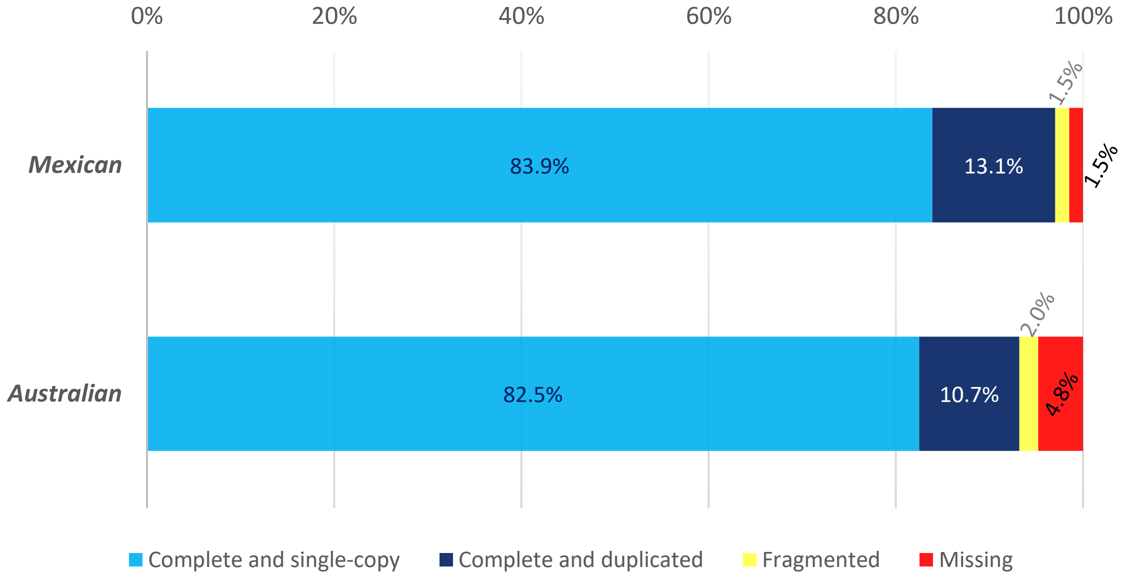

Supplementary Figure S8. <u>DNA methylation context identified in *S. hispanica* genome</u>. **a**) Global DNA methylation percentage breakdown by context (CG, CHG and CHH). **b**) Representation of gene body methylation in all contexts 1000 bp upstream and downstream. **c**) Representation TE body methylation in all contexts 1000bp upstream and downstream.

a

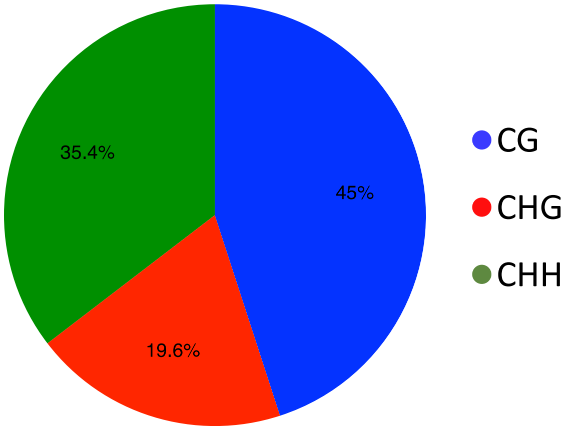

b

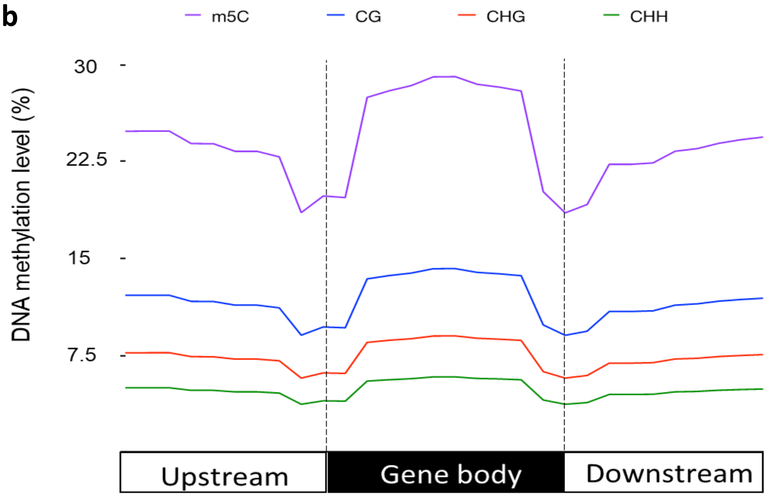

c

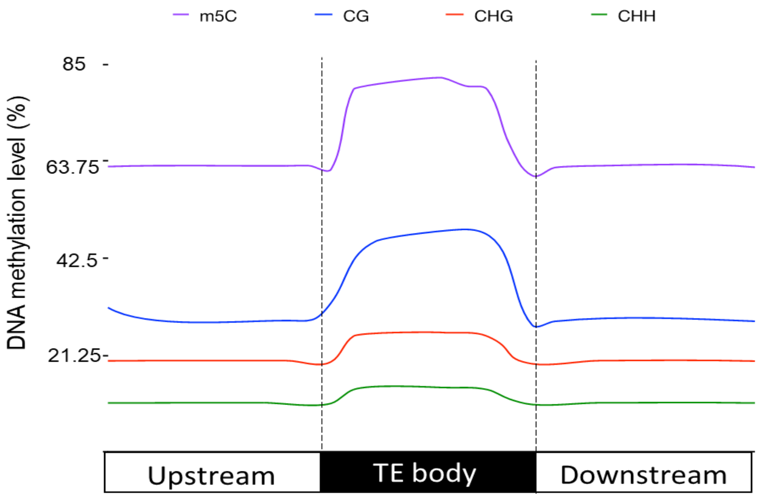

Supplementary Figure S9. Gene, repetitive sequences, and methylation density in *S. hispanica* chromosomes. Chromosomes show high gene density (green) in regions with low level of repetitive DNA (gray). Centromeres (black rows) were identified in genomic regions with high levels of methylation (red).

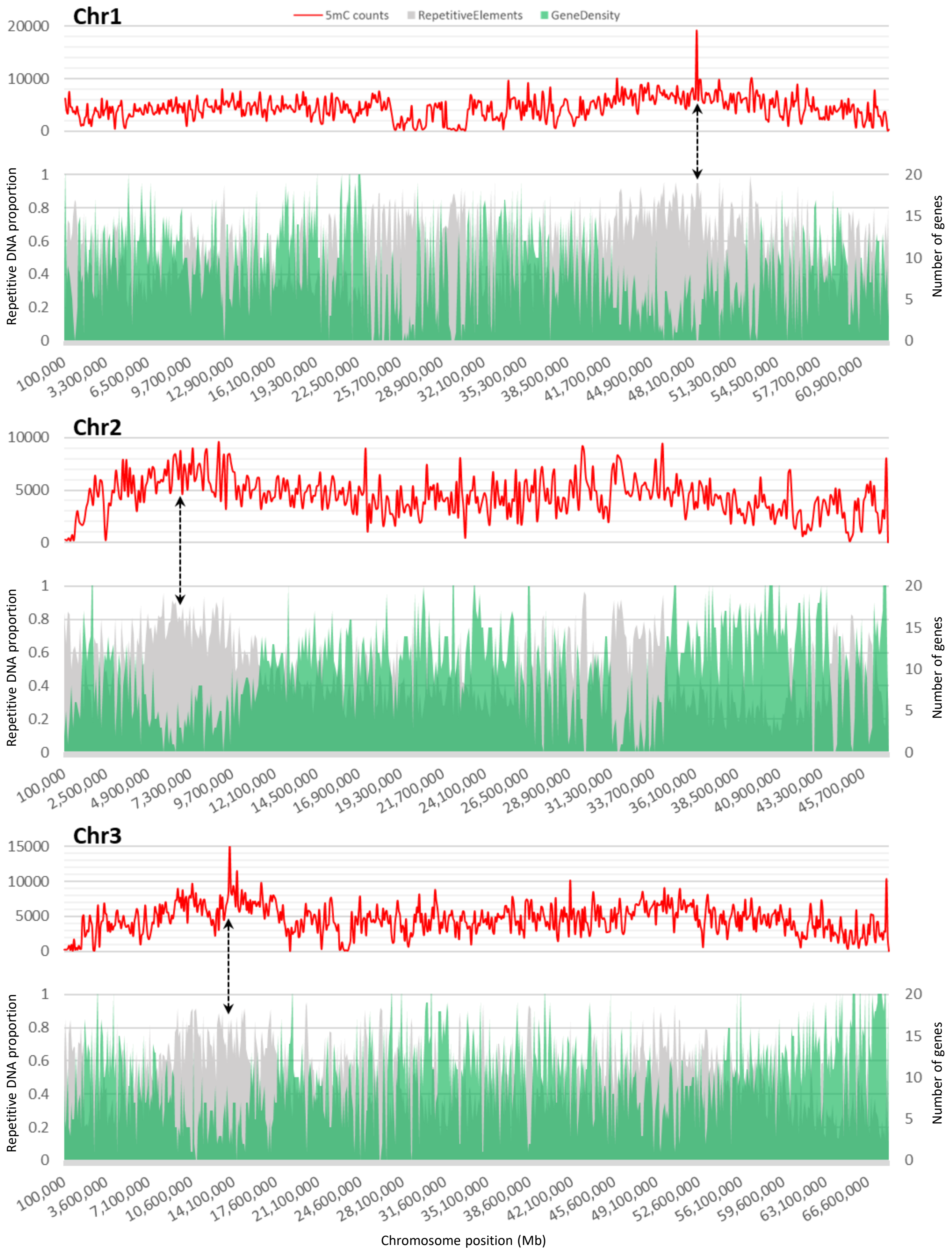

Supplementary Figure S9 (continued). Gene, repetitive sequences, and methylation density in *S. hispanica* chromosomes. Chromosomes show high gene density (green) in regions with low level of repetitive DNA (gray). Centromeres (black rows) were identified in genomic regions with high levels of methylation (red).

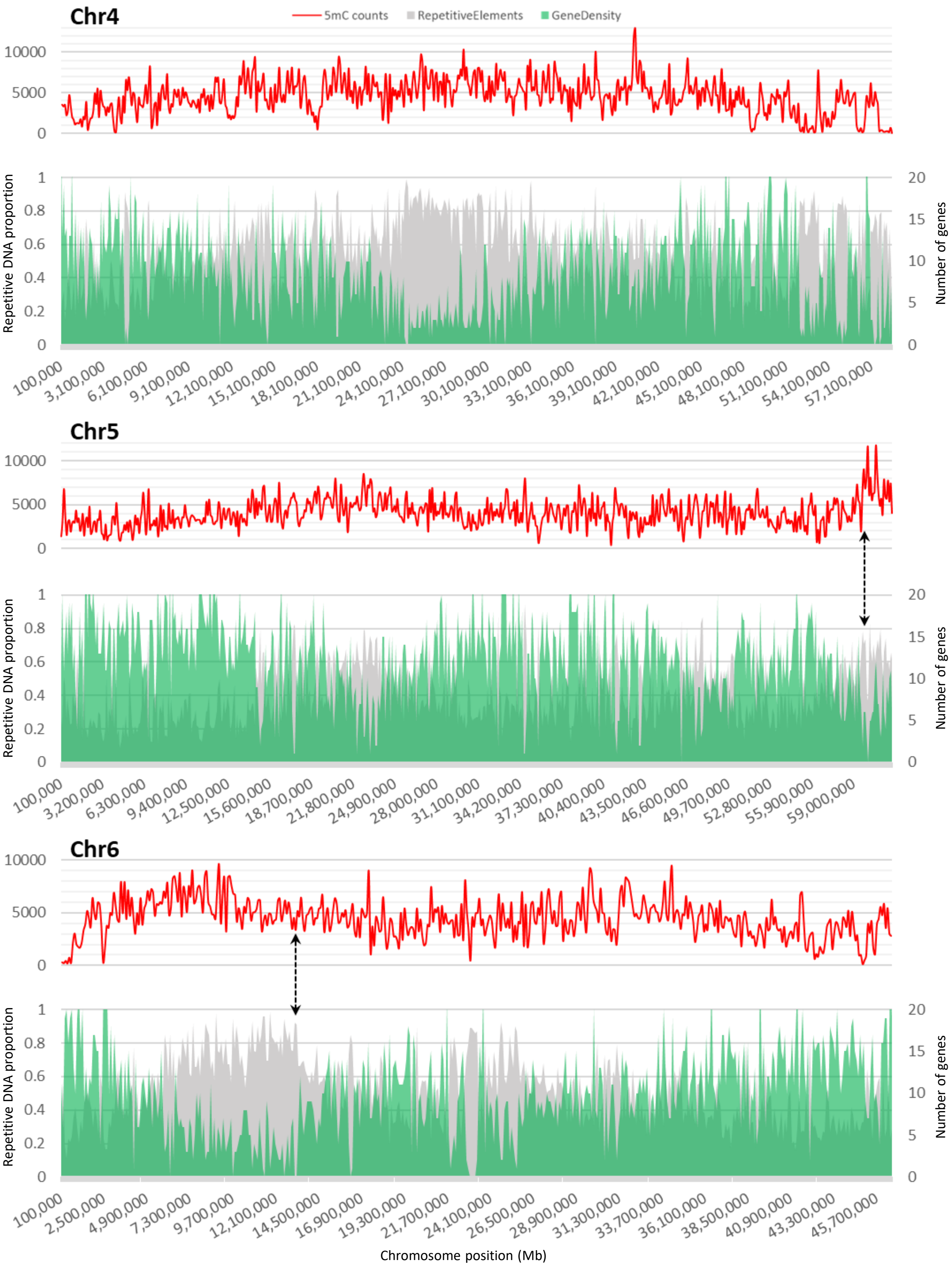

Supplementary Figure S10. Heatmap showing expression of *S. hispanica* genes in different tissues and stages. Expression profiles (*z* score normalized) across different tissues during the life cycle of *S. hispanica*. Detailed description of the plant material in Supplementary Table S8.

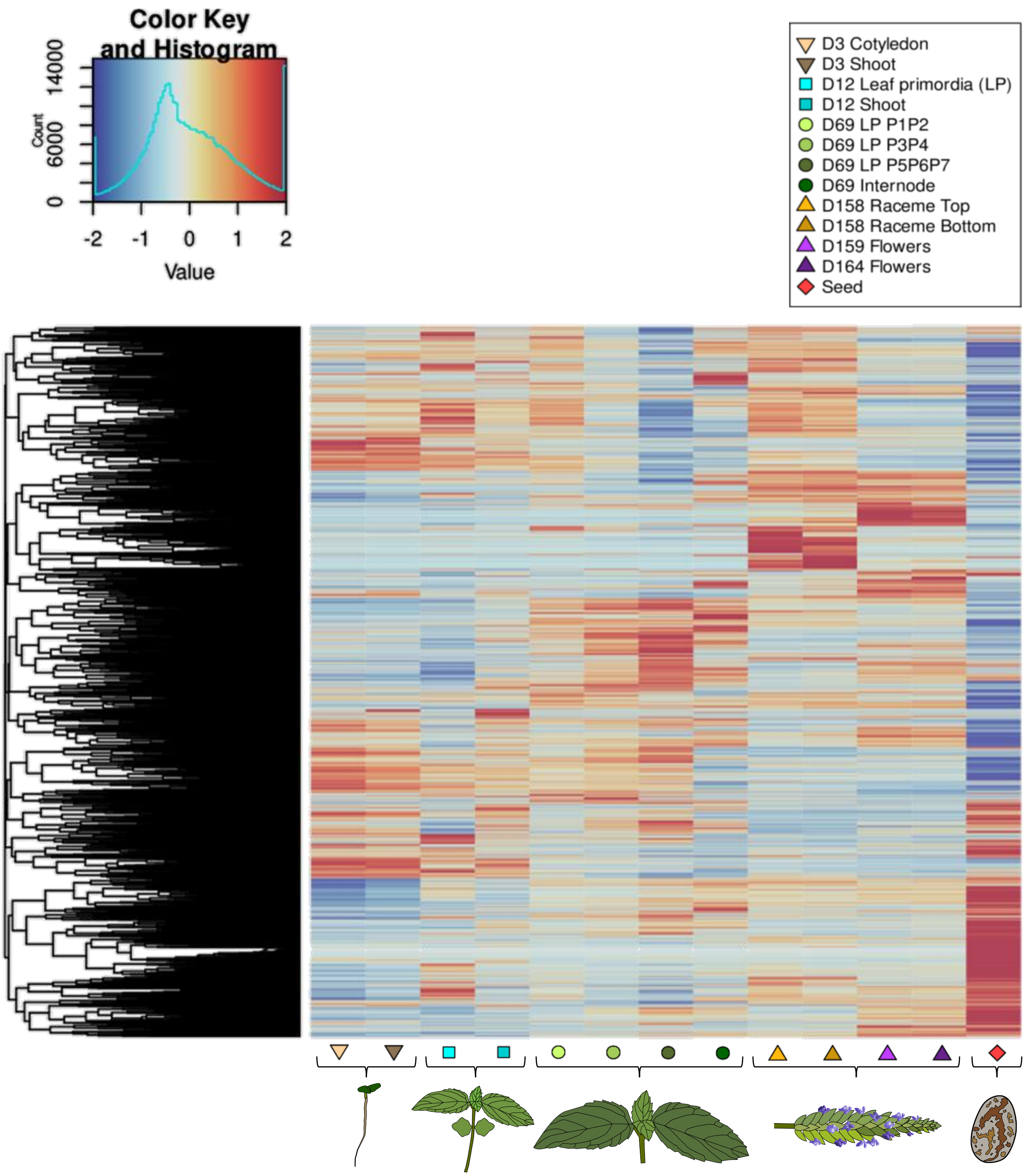

Supplementary Figure S11. Gene ontology (GO) enrichment analysis for genes highly expressed in *S. hispanica* seeds. GO biological process enriched for genes with maximum expression in seed (Supplementary Fig. S8). Circle color indicates the proportion of genes with maximum expression in seed over the number of genes in each category. Enrichment of GO terms was conducted using topGO. Cut-off lines drawn at equivalents of *p*=0.05, *p*=0.01, *p*=0.001.

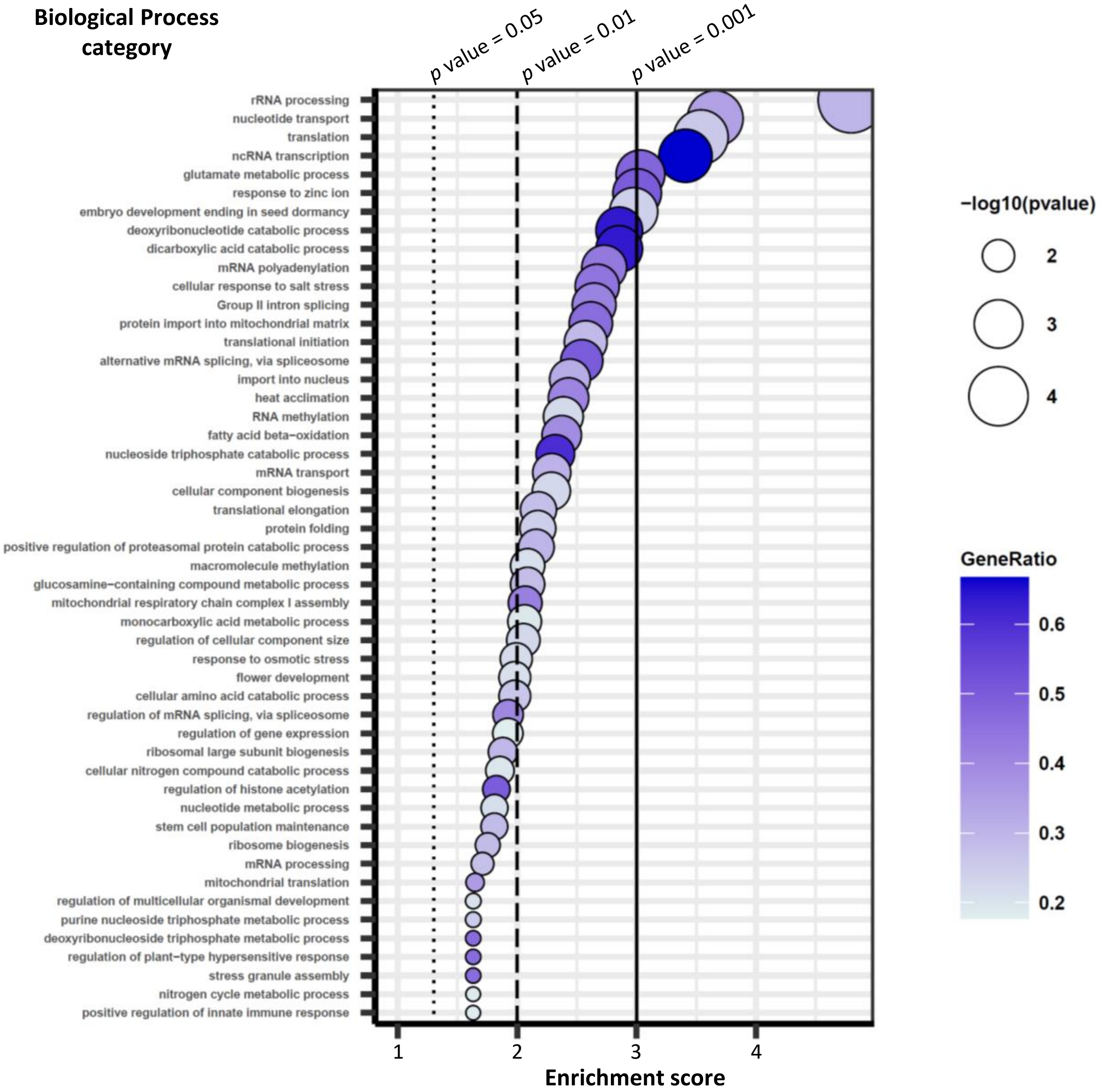

Supplementary Figure S12. Lipidic composition of chia seeds. **a**) Major classes of lipids identified in *S. hispanica* dry seeds. **b**) Detail of the most abundant single lipids in the triacylglycerols (TG) class. Diacylglycerols, DG; fatty acids, FA; lysophosphatidylcholine, LPC; monoacylglycerols, MG; phosphatidylcholine, PC; phosphatidylethanolamine, PE; phosphoglycerols, PG; phosphatidylinositols, PI; sitoesteryl ester, SiE.

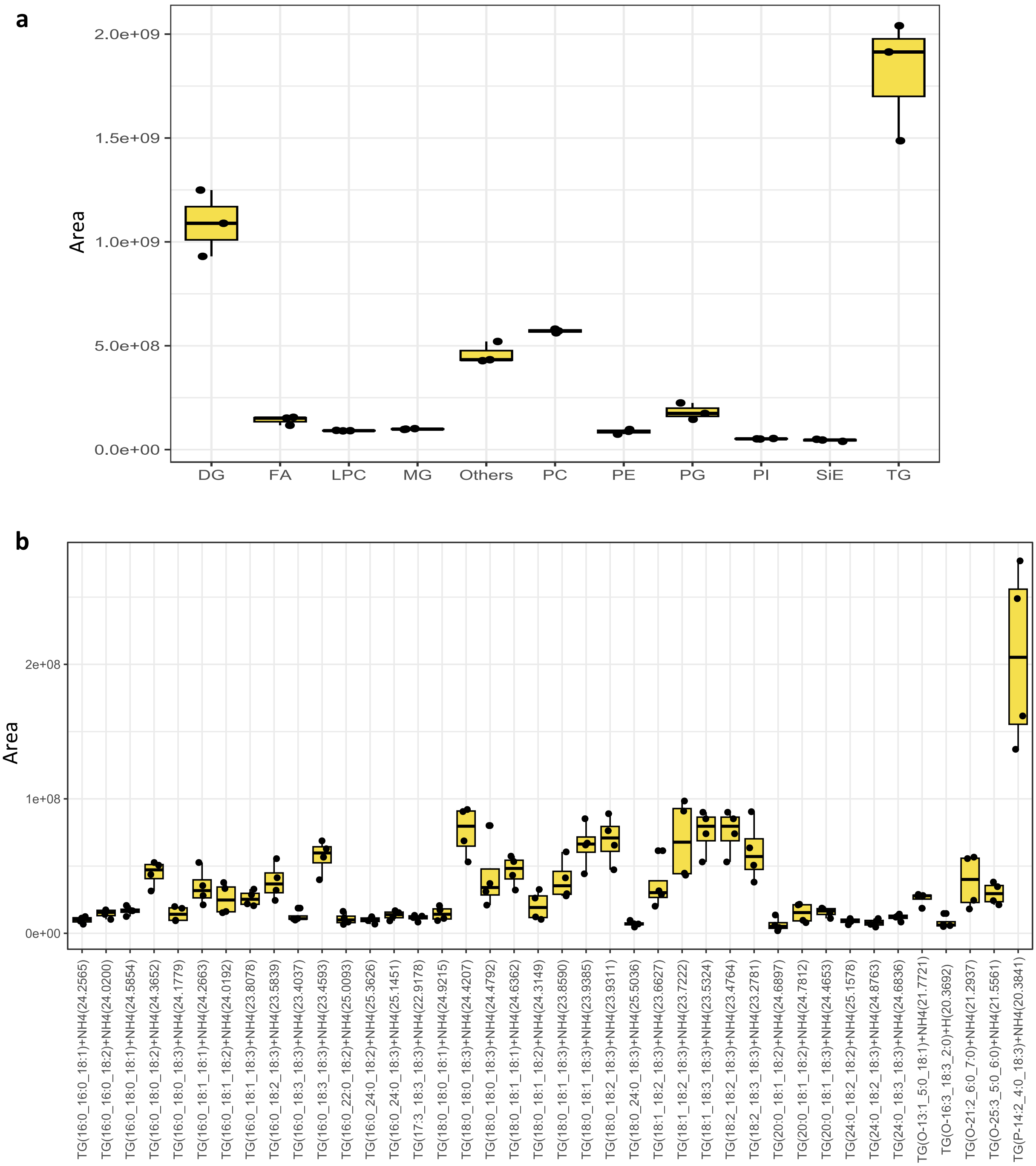

Supplementary Figure S13. Mucilage-related genes identified in *S. hispanica* genome. Genes involved in seed mucilage metabolism in *Arabidopsis thaliana* were identified in *S. hispanica* genome. Their proposed or reported function is indicated by a color code (bottom).

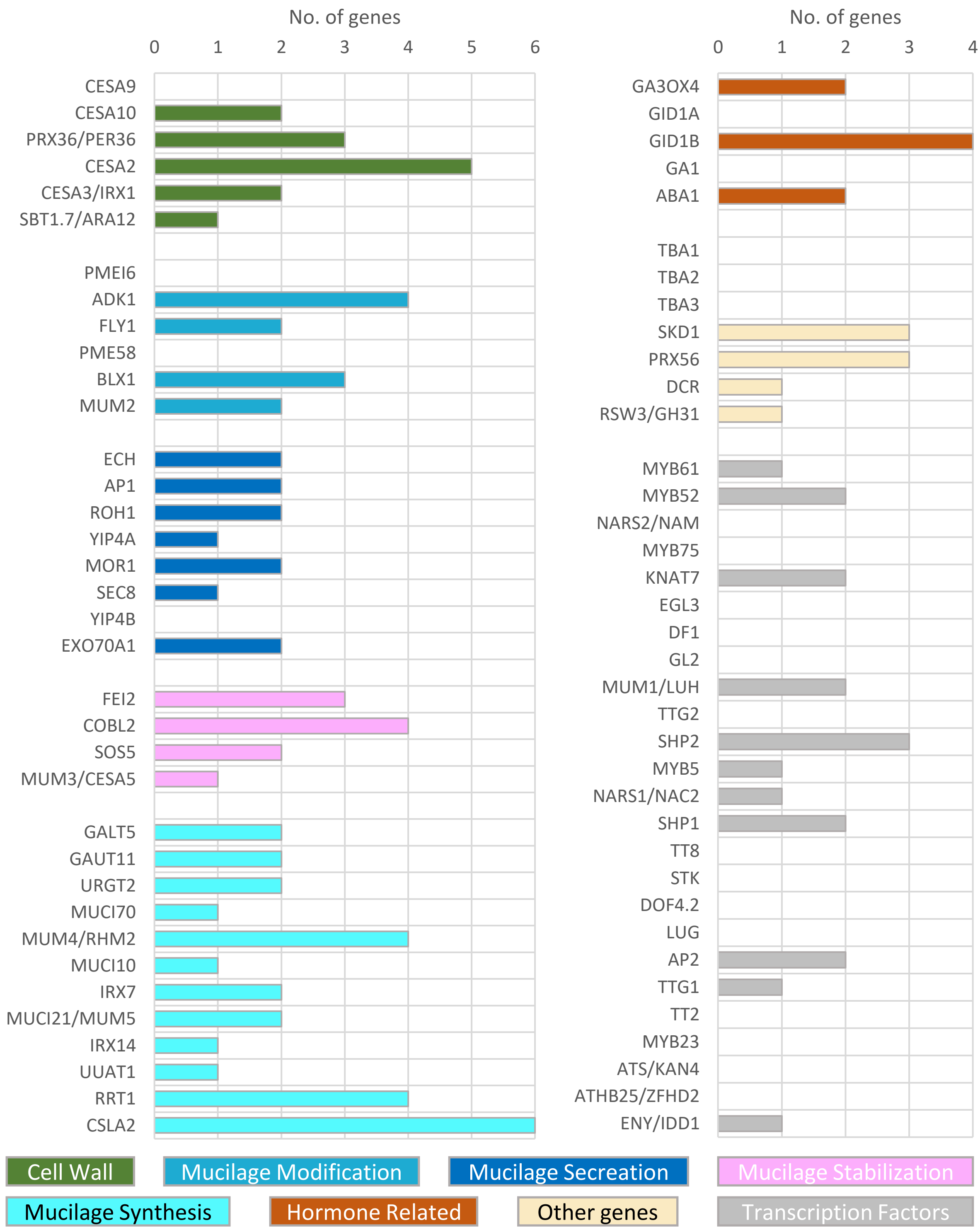

Supplementary Figure S14. Heatmap showing gene expression of the complete set of proteins in mucilage sample during seed development. This analysis included the complete set of proteins (95 proteins) present in the chia mucilage sample. Expression profiles (*z* score normalized) during seed development at specific days after flowering (DAF). Detailed description of the plant material in Supplementary Table S8.

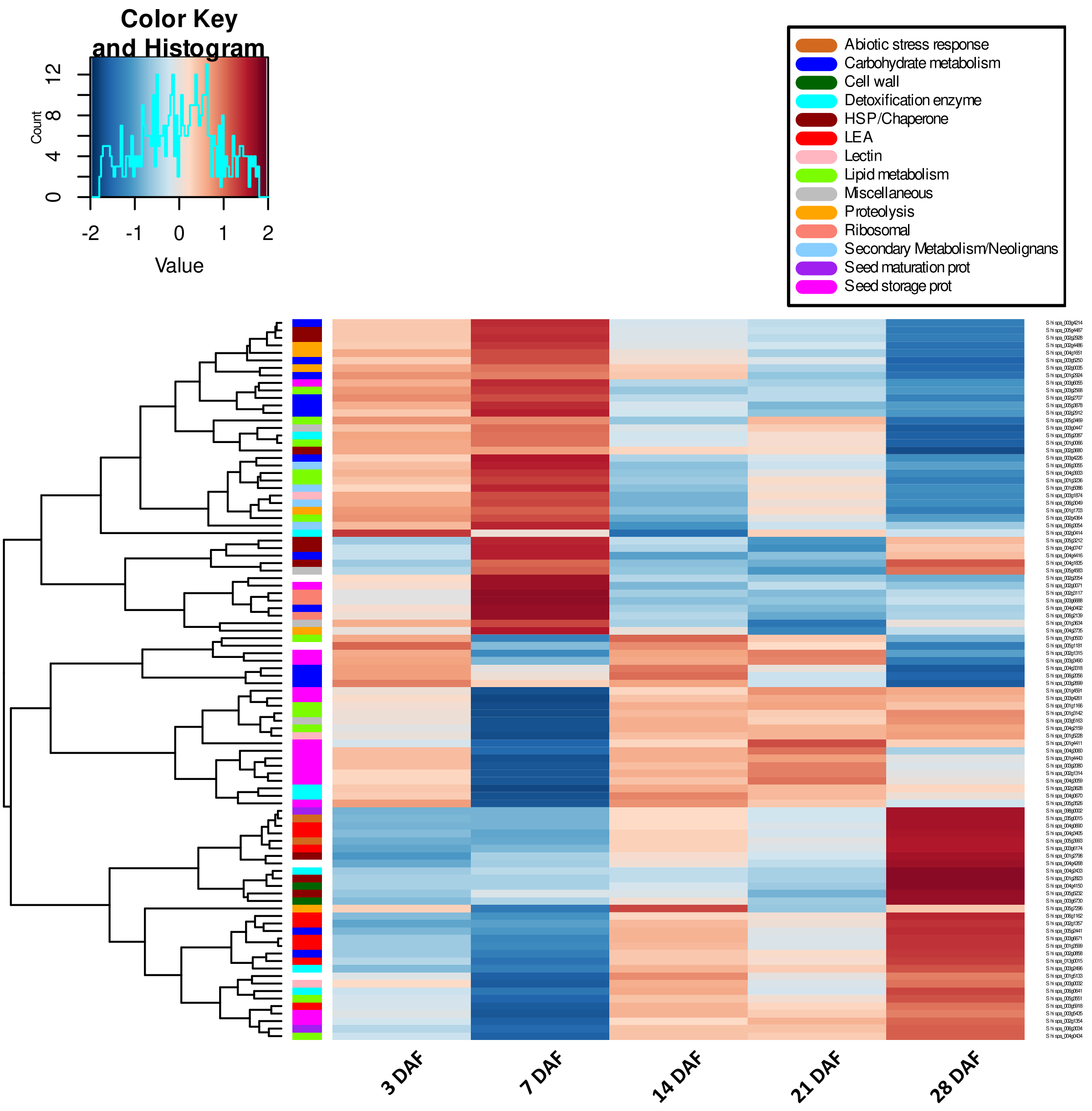

Supplementary Figure S15. Expression pattern of mucilage metabolism regulators during chia seed development. Transcription factors (TFs) probably involved in *S. hispanica* mucilage production identified by the regulatory network analysis. Gene expression is shown as $\log_2$(TPM) at different days after flowering (DAF). TFs which *Arabidopsis* homologs have a reported function in mucilage production are indicated in red.

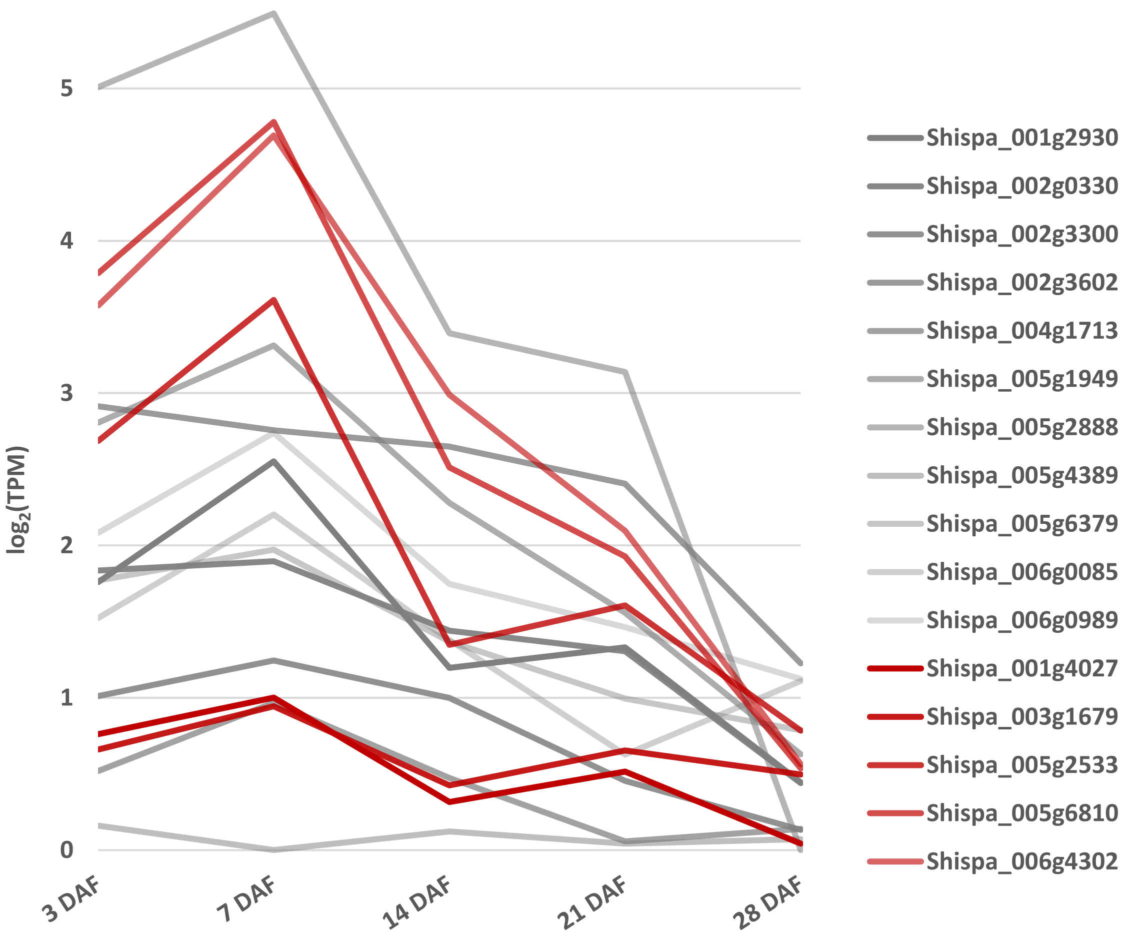

Supplementary Figure S16. <u>Preliminary results of embryo development characterization in *S. hispanica*</u>. Chia flowers were collected at 3 days after anthesis and fixed in 95% ethanol:glacial acetic acid (3:1) for 30 minutes at room temperature. After fixation the ovules were dissected and stored in 70% ethanol at -20° C until examined. For microscopic visualization, the ovules were cleared and softened on 0.8 M NaOH overnight at room temperature. Ovules were mounted in 50% 0.1 M K_2PO_4 and 50% glycerol and observed in a clear field using an Olympus IX73 microscope. This analysis showed that this time correlates with early-heart stage of the *Arabidopsis* embryo development. Scale bar: 100 µm

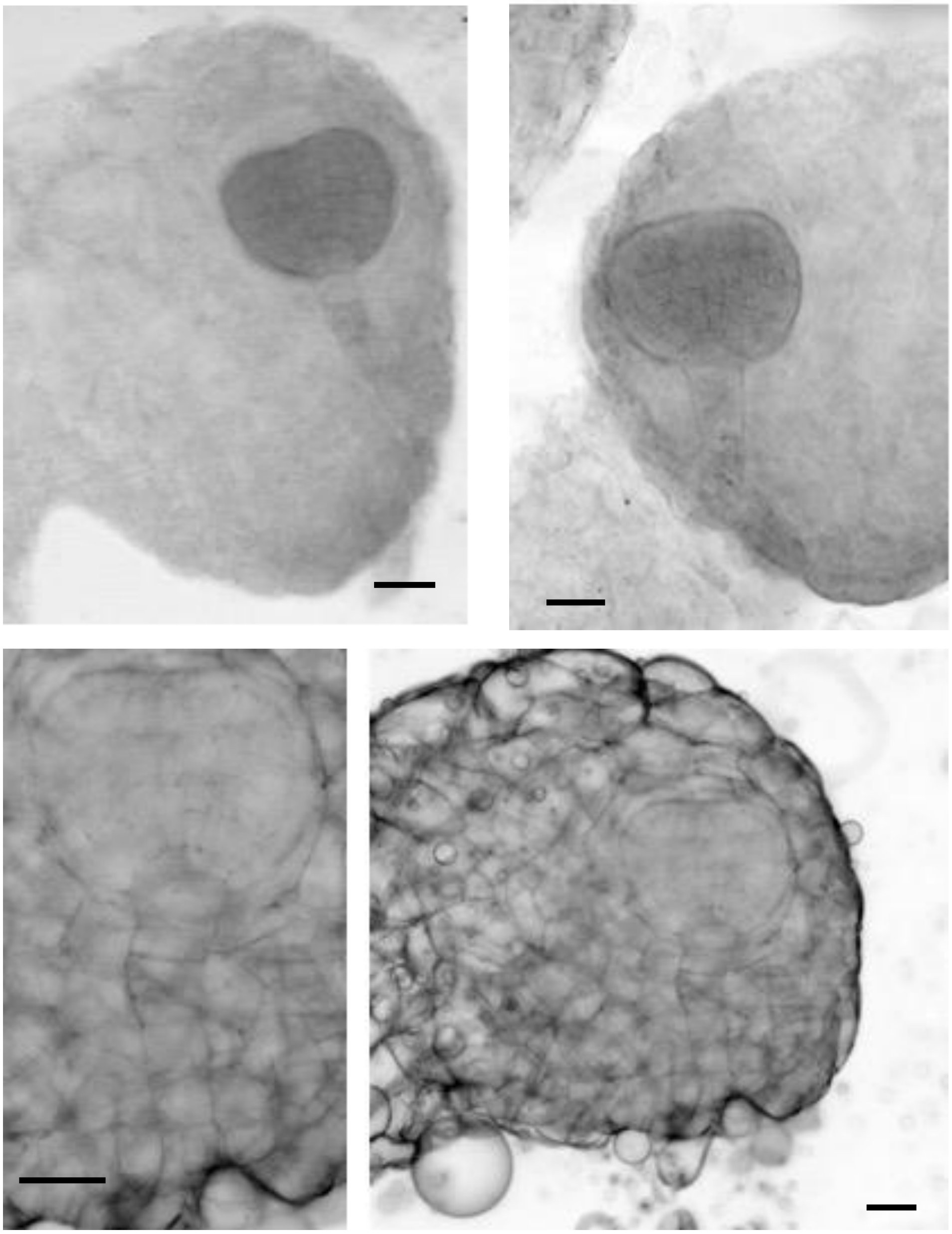

SUPPLEMENTARY TABLES

Supplementary Table S1. Summary of *S. hispanica* genome sequencing data.

**Sequencing platform**	**Raw data (bases)**	**Estimated coverage[a]**
ONT ultra-long reads	46,687,679,351	130.29×
Illumina PE150 reads	54,870,648,900	153.13×
Hi-C sequencing	25,132,794,600	70.17×
**TOTAL**	**126,691,122,851**	**353.59×**

[a]Overall coverage estimated on a genome size of 358 Mb.

Supplementary Table S2. Evaluated pipelines to generate a preliminary *S. hispanica* genome assembly using ONT reads.

	**Asembler**			
**Features**	SMARTdenovo[a]	Flye	NECAT	raven
Total contigs	416	1,142	**269**	1,290
#contigs (>= 50 kb)	382	613	232	1,085
N50	2,041,060	1,742,496	**5,378,029**	519,181
Largest contig	11,047,416	12,961,729	**29,785,949**	3,932,890
Total length	371,449,452	370,719,619	372,986,686	365,954,445
GC (%)	36.64	37.04	37.04	36.44
BUSCO (lineage dataset: Embryophyta)				
Complete BUSCOs	75.3%	97.4%	**97.8%**	97.7%
Complete and single-copy	70.0%	84.0%	83.7%	85.4%
Complete and duplicated	5.3%	13.4%	14.1%	12.3%
Fragmented	10.7%	1.2%	1.0%	1.1%
Missing	13.9%	1.4%	**1.2%**	**1.2%**

[a]Additional consensus step using racon.

Supplementary Table S3. Comparison of the *S. hispanica* genome assembly generated in this study and a previous genome version[a].

	***S. hispanica* genome**	
**Features**	Mexican variety	Australian variety
Total No. of sequences	154	171
Sequences >= 50kb	50	23
Total length	351,989,704	347,748,913
Largest scaffold	68,315,700	68,283,714
N50	61,893,606	57,928,046
L50	3	3
GC (%)	36.61%	36.59%
# N's per 100 kbp	3.44	55.07
Predicted gene models	34,748	31,069

The metrics of the genome reference generated in this study (Mexican variety) is compared to the previously reported chia genome (Australian variety; Wang *et al.*, 2022).

Supplementary Table S4. BUSCO analysis of genome assembly.

	**Lineage dataset**	
	Embryophyta	Eudicots
Complete BUSCOs	98.5%	97.1%
Complete and single-copy BUSCOs	86.2%	83.0%
Complete and duplicated BUSCOs	12.3%	14.1%
Fragmented BUSCOs	0.4%	0.5%
Missing BUSCOs	1.1%	2.4%
Total BUSCO groups searched	1,614	2,326

Supplementary Table S5. Comparison of the chromosome sizes between the genome reference generated in this study (Mexican variety) and a previously reported chia genome (Australian variety; Wang *et al.*, 2022).

**Chromosome**	**Mexican variety**	**Australian variety**
Chr 1	63,063,703	57,928,046
Chr 2	47,121,767	51,386,454
Chr 3	68,315,700	68,283,714
Chr 4	58,534,600	57,656,806
Chr 5	61,893,606	61,825,500
Chr 6	46,599,015	47,361,193
Assembly anchored in chromosomes	345,528,391	344,441,713

Supplementary Table S6. Proposed positions for centromeres in the *S. hispanica* genome.

Chromosome	Start position	End position
Chr 1	48,569,070	48,622,805
Chr 2	6,709,596	6,784,369
Chr 3	13,758,850	13,785,444
Chr 4	---	---
Chr 5	59,944,408	60,056,950
Chr 6	13,278,126	13,294,695

Start and end positions were only delimited by the centromeric repeats indicated in Supplementary Table 7.

Supplementary Table S7. Consensus sequence of the most common centromeric repeats.

>Centromeric_repeat_1 (171 bp)<br>AAACCTATTGAATAGAAATTGCCCACGACCATGCATTTTGTGATGTTAGTAACGCAACGACAATGAAGAAAAGATCATTTTGGT<br>CAATAGAGTATGATAATCGTTGTTGTTTCTCGTGCACGTTCATTTAAAGCTTAACTCTTTATATTTGGGTTTACTTGGCCAACAAG<br>C
>Centromeric_repeat_2 (171 bp)<br>GTGGGCAATTTCTATTCAATAGGTTTGCTTGTTGGCCAAGTAAACCCAAATATAAAGAGTTAAGCTTTAAATGAACGTGCACGA<br>GAAACAACAACGATTATCAAACTCTATTGACCAAAATGATCTTTTCTTCATTGTCGTTGCGTTACTAACATCACAAAATGCATGG<br>TC
>Centromeric_repeat_3 (174 bp)<br>AAAATGTTGTCAAAACAAGGATAACTCTACTTATATGCGTTTAGAAAACCCTAAAAACTCAAAAAAAAAANNNAAANNANAA<br>AAAAAGAATAGAAATAAAAAAAAAACCAAAAAAAGAGTAAATTATCTTTTTCGCCCCTAAACGATGTGAAGTGGCCAAAATTC<br>GTCGTCCAA
>Centromeric_repeat_4 (167 bp)<br>GTGGGAAATTTTATTTGAATTGGTATGCTTGTTGGCCAAGTAAACCAAAATATAAAGATTTAAGCTTTAAATGAACTTTTACGAG<br>ATAACAATGATTAATATGCTCTATTGACCAAAATGATCTTGTCTTGAATGTCGTTTTTTTACAAAGCATCCTATGTATGTGA

Supplementary Table S8. Description of RNA-seq data and plant material included in the gene expression analysis.

Sample ID	Sample description	Time[a]	Samples description, replicates and reference
Seed	Dry seed	0 DAS	Chia life cycle<br><br>3 replicates per sample<br><br>Gupta *et al.* (2021)
D3 Cotyledon	Green cotyledon	3 DAS	
D3 Shoot	Above ground shoot		
D12 Shoot	Above ground shoot	12 DAS	
D12 LP	Youngest leaf at the shoot apex		
D69 LP P1P2	1st and 2nd leaves at the shoot apex	69 DAS	
D69 LP P3P4	3rd and 4th leaves at the shoot apex		
D69 LP P5P6P7	5th, 6th and 7th leaves at the shoot apex		
D69 Internode	Internode between 5th and 6th leaves		
D158 Raceme-T	Raceme inflorescence (top half)	158 DAS	
D158 Raceme-B	Raceme inflorescence (bottom half)		
D159 Flowers	Flowers from Day-1 of flowering	159 DAS	
D164 Flowers	Flowers from Day-5 of flowering	164 DAS	
Seed 3d	Developing seeds	3 DAF	Chia seed development<br><br>No replicates<br><br>Sreedhar *et al.* (2015)
Seed 7d	Developing seeds	7 DAF	
Seed 14d	Developing seeds	14 DAF	
Seed 21d	Developing seeds	21 DAF	
Seed 28d	Completely mature embryo	28 DAF	

[a]Expressed as Days After Sowing (DAS), or Days After Flowering (DAF). LP, Leaf primordia.

Supplementary Table S9. Curated metabolome of most abundant chromatographic peaks in dry *S. hispanica* seeds.

Tags	Name	RT [min]	Rel. area[a]	Reference Ion
Polyphenol	(R)-(+)-rosmarinic acid	14.131	0.263467	[M-H]-1
Fatty acid	Oleamide	21.156	0.232611	[M+H]+1
Carbohydrate	α,α-Trehalose	2.001	0.225578	[M-H]-1
Other	3-hydroxycoumarin	13.57	0.221071	[M+H]+1
Organic acid	THREONIC ACID, L-	2.044	0.103275	[M-H+HAc]-1
Carbohydrate	stachyose	1.811	0.070159	[M+FA-H]-1
Other	Buchananine	1.922	0.068051	[M+H]+1
Other	L- α-Glycerylphosphorylcholine	1.818	0.065639	[M+H]+1
Fatty acid	α-Linolenic acid	20.919	0.044953	[M-H]-1
Amino Acid	L-Phenylalanine	8.003	0.044255	[M+H]+1
Fatty acid	Palmitoleic Acid	19.16	0.043417	[M+H]+1
Carbohydrate	raffinose	2.034	0.043116	[M+FA-H]-1
Carbohydrate	beta-Maltose	2.017	0.039673	[M+K]+1
Fatty acid	Linoleamide	20.634	0.035778	[M+H]+1
Nucleotide	Adenosine	4.435	0.025639	[M+H]+1
Amino Acid	L-Arginine	1.548	0.025496	[M+H]+1
Fatty acid	Stearidonic acid	19.605	0.025104	[M+H]+1
Fatty acid	(9E)-11-{3-[(2Z)-2-Penten-1-yl]-2-oxiranyl}-9-undecenoic acid	19.154	0.0251	[M-H]-1
Fatty acid	[Similar to: (±)9-HpODE; ΔMass: -0.1670 Da]	14.138	0.022347	[M+FA-H]-1
Carbohydrate	L-Iditol	1.842	0.020105	[M-H]-1
Amino Acid	L-(+)-Valine	1.833	0.017268	[M+H]+1
Other	12-O-Î²-D-Glucopyranosyloxyjasmonic acid	12.255	0.016802	[M+Na]+1
Amino Acid	L-Glutamic acid	1.815	0.016697	[M+H]+1
Fatty acid	α-Eleostearic acid	19.472	0.016197	[M+H]+1
Carbohydrate	D-(+)-Maltose	2.057	0.015637	[M+H]+1
Amino Acid	Asparagine	1.735	0.014689	[M+H]+1
Polyphenol	Cinnamic acid	8.063	0.014302	[M+NH4]+1
Amino Acid	L-Pyroglutamic acid	3.282	0.013736	[M+H]+1
Carbohydrate	Maltotriose	2.058	0.01301	[M+K]+1
Fatty acid	(+/-)9(10)-EpOME	19.483	0.012993	[M-H]-1
Fatty acid	Linoleic Acid	21.435	0.01268	[M-H]-1
Polyphenol	2-Hydroxycinnamic acid	13.787	0.012439	[M+H+MeOH]+1
Fatty acid	Azelaic acid	14.939	0.011376	[M-H]-1
Carbohydrate	bis-beta-D-fructofuranose 1,2':2,3'-dianhydride	1.845	0.011344	[M+H]+1
Carbohydrate	1-(sn-glycero-3-phospho)-1D-myo-inositol	2.168	0.01096	[M-H]-1
Carbohydrate	1-deoxy-D-altro-heptulose 7-phosphate	2.172	0.01096	[M-H+HAc]-1
Amino Acid	L-Tyrosine	3.421	0.010646	[M+H]+1
Fatty acid	(±)9-HpODE	18.527	0.009978	[M-H]-1
Other	2,5-Dihydroxybenzoate 2-O-β-D-glucoside	9.562	0.008449	[M-H]-1
Amino Acid	4-Oxoproline	3.269	0.008368	[M-H]-1

Fatty acid	cis-6,7-epoxystearic acid	19.793	0.007275	[M-H]-1
Carbohydrate	Sucrose	1.751	0.007249	[M-H]-1
Organic acid	Gluconic acid	2.225	0.00708	[M-H]-1
Nucleotide	Guanosine	5.455	0.006466	[M-H]-1
Carbohydrate	2-(α-D-mannosyl)-D-glyceric acid	2.036	0.006215	[M-H]-1
Nucleotide	Uridine	3.497	0.005931	[M-H]-1
Amino Acid	L-Glutamic acid	1.78	0.005769	[M-H]-1
Carbohydrate	2,3,4,5-Tetrahydroxypentanal	2.313	0.005732	[M+Cl]-1
Fatty acid	Oleic acid	22.138	0.005626	[M-H]-1
Fatty acid	Myristic Acid	20.993	0.005407	[M-H]-1
Polyphenol; Organic acid	Lactobionic acid	1.956	0.004854	[M-H]-1
Organic acid	3-Ureidopropionic acid	1.717	0.004274	[M-H]-1
Fatty acid	13(S)-HpOTrE	19.2	0.003936	[M-H]-1
Fatty acid	NP-020521	19.837	0.003448	[M-H]-1
Org. acid	Succinic acid	3.95	0.003351	[M-H]-1
Carbohydrate	Hex-2-ulose	2.01	0.003294	[M-H]-1
Fatty acid	13(S)-HOTrE	19.504	0.00306	[M-H]-1
Nucleotide	Xanthosine	8.189	0.002778	[M-H]-1
Carbohydrate	Hept-2-ulose	2.152	0.002291	[M-H]-1
Polyphenol	curcumin II	14.693	0.001162	[M-H]-1
Amino Acid	DL-TYROSINE	3.432	0.000939	[M-H]-1
Organic Acid	4-[(1S,3aR,4S,6aR)-4-(4-hydroxy-3-methoxyphenyl)-hexahydrofuro[3,4-c]furan-1-yl]-2-methoxyphenol	12	0.000356	[M+H]+1
Polyphenol	Asperglaucide	17.874	0.000144	[M+H]+1

[a]Measured as normalized area under each chromatographic peak.

Supplementary Table S10. Complete list of *S. hispanica* candidate genes for rosmarinic acid biosynthesis.

Rxn	EC number[a]	*S. hispanica* genes
A1	2.6.1.5	Shispa_002g1931, Shispa_002g2389, Shispa_002g4689, Shispa_004g2493, Shispa_004g4814, Shispa_004g4815, Shispa_005g5008, Shispa_005g7480, Shispa_006g4391, Shispa_006g4392, Shispa_006g4393, Shispa_006g4394, Shispa_006g4395, Shispa_006g4398, Shispa_006g4399, Shispa_006g4400
A2	1.1.1.237	Shispa_002g2559, Shispa_002g4310, Shispa_006g1350
B1	4.3.1.24[b]	Shispa_002g0701, Shispa_003g0648, Shispa_003g4136, Shispa_005g1688, Shispa_005g4047, Shispa_006g2148
B2	1.14.13.11	Shispa_001g3896, Shispa_005g2309, Shispa_005g4261, Shispa_005g4262, Shispa_005g4263
B3	6.2.1.12	Shispa_001g0531, Shispa_002g1714, Shispa_004g1257, Shispa_004g1496, Shispa_004g1497, Shispa_004g2430, Shispa_004g4958, Shispa_005g4525, Shispa_006g1407, Shispa_006g3516, Shispa_051g0005
C1	2.3.1.140[c]	Shispa_001g0114, Shispa_001g0132, Shispa_001g0137, Shispa_001g0187, Shispa_001g1090, Shispa_001g4427, Shispa_001g4428, Shispa_001g4429, Shispa_001g4945, Shispa_001g5174, Shispa_002g0167, Shispa_002g0369, Shispa_002g0377, Shispa_002g1242, Shispa_002g3004, **Shispa_002g3426**, Shispa_002g3427, **Shispa_002g3428**, Shispa_003g0520, Shispa_003g0525, Shispa_003g0526, Shispa_003g0528, Shispa_003g0999, Shispa_003g1079, Shispa_003g4585, **Shispa_003g4881**, Shispa_003g4903, Shispa_003g4905, **Shispa_003g4911**, Shispa_003g4964, Shispa_003g5835, Shispa_003g6499, Shispa_003g6500, Shispa_004g0322, Shispa_004g0524, Shispa_004g0525, Shispa_004g0527, Shispa_004g0739, Shispa_004g0740, Shispa_004g1138, Shispa_004g3427, Shispa_005g0527, Shispa_005g1008, Shispa_005g1506, Shispa_005g2005, Shispa_005g3016, Shispa_005g3571, Shispa_005g5223, Shispa_005g6236, Shispa_006g0194, **Shispa_006g1185**, Shispa_006g2371, Shispa_006g2570, Shispa_006g2590, Shispa_006g3636, Shispa_006g3639, Shispa_006g3640
D1<br>D2<br>E1<br>E2	1.14.14.-	Shispa_001g0113, Shispa_001g0115, Shispa_001g0116, Shispa_001g0117, Shispa_001g0119, Shispa_001g0121, Shispa_001g0753, Shispa_001g0754, Shispa_001g1061, Shispa_001g1062, Shispa_001g1065, Shispa_001g1066, Shispa_001g3769, Shispa_001g5034, Shispa_002g2953, **Shispa_002g3485**, Shispa_003g1648, Shispa_003g1649, Shispa_003g1650, Shispa_003g1837, Shispa_003g1841, Shispa_003g1842, Shispa_003g2645, Shispa_003g2959, Shispa_003g3771, Shispa_003g4901, Shispa_003g5289, Shispa_004g0009, Shispa_004g0516, Shispa_004g0517, Shispa_004g0518, Shispa_004g0519, Shispa_004g0520, Shispa_004g0773, Shispa_004g0774, Shispa_004g1174, Shispa_004g2368, Shispa_004g2370, Shispa_004g2854, Shispa_005g1682, Shispa_005g2796, Shispa_005g2797, Shispa_005g5162, Shispa_005g5306, Shispa_005g6298, Shispa_005g6299, Shispa_005g6302, Shispa_005g7208, Shispa_006g2752, Shispa_006g2753, Shispa_006g3991, Shispa_031g0001, **Shispa_031g0008**, Shispa_033g0004, Shispa_033g0005, Shispa_062g0004

[a]Reaction EC number (Rxn) described in Trócsányi, *et al.* (2020). Genes with maximum expression in *S. hispanica* seed (blue). [b]Similar to Phenylalanine ammonia-lyase (EC 4.3.1.5). [c]Homologs of *S. miltiorrhiza* and *M. officinalis* rosmarinic acid synthase (GenBank: FJ906696.1 and FR670523.1, respectively).

Supplementary Table S11. Mucilage-related genes in *S. hispanica* genome.

Category	*S. hispanica* genes
Cell wall	Shispa_001g1243, Shispa_002g2780, Shispa_003g2536, Shispa_003g4636, Shispa_003g5816, Shispa_004g2161, Shispa_005g0341, Shispa_005g2402, Shispa_005g2429, Shispa_005g6061, Shispa_005g7266, Shispa_005g7304, Shispa_006g2491
Hormone related	Shispa_001g1144, **Shispa_001g4024**, Shispa_002g1403, Shispa_002g2494, Shispa_003g6801, Shispa_006g0825, Shispa_006g1881, Shispa_027g0002
Mucilage modification	Shispa_001g3639, Shispa_002g0685, Shispa_002g1446, Shispa_002g2912, Shispa_003g5720, Shispa_004g0595, Shispa_004g4825, Shispa_005g2128, Shispa_005g4045, Shispa_006g4412, Shispa_017g0001
Mucilage secretion	Shispa_001g2024, Shispa_002g0915, Shispa_002g3381, Shispa_003g0976, Shispa_003g1641, Shispa_003g3913, Shispa_004g1258, Shispa_004g1993, Shispa_005g0048, Shispa_005g5646, Shispa_005g6887, Shispa_006g0418
Mucilage stabilization	Shispa_001g1277, Shispa_002g3826, Shispa_003g1225, Shispa_003g5390, Shispa_004g3011, Shispa_005g0649, Shispa_005g1885, Shispa_005g5780, Shispa_006g1459, Shispa_006g4081
Mucilage synthesis	Shispa_001g1606, Shispa_001g2227, Shispa_001g3258, Shispa_002g0640, Shispa_002g1112, Shispa_002g1127, Shispa_002g1435, Shispa_002g3856, Shispa_002g4640, Shispa_003g2232, Shispa_003g2824, Shispa_003g2976, **Shispa_003g4211**, **Shispa_003g4212**, Shispa_003g5495, Shispa_004g2191, Shispa_004g2456, Shispa_004g3466, Shispa_005g0967, Shispa_005g1296, Shispa_005g1490, Shispa_005g1985, Shispa_005g2017, Shispa_005g3032, **Shispa_005g3659**, **Shispa_005g3663**, Shispa_005g5181, Shispa_006g2413
Other genes	Shispa_003g5165, Shispa_004g4869, Shispa_004g4994, **Shispa_005g1454**, **Shispa_005g1455**, Shispa_005g3845, Shispa_006g2590, Shispa_006g4485
Transcription factors	Shispa_001g3018, Shispa_001g3078, **Shispa_001g4027**, Shispa_002g1478, Shispa_003g1037, Shispa_003g1679, Shispa_003g5229, Shispa_004g3362, Shispa_004g4724, Shispa_005g2533, Shispa_005g2888, Shispa_005g3080, Shispa_005g4789, Shispa_005g6810, Shispa_006g0183, Shispa_006g2026, Shispa_006g4113, Shispa_006g4302

Tandem gene duplications are indicated in **bold**.

Supplementary Table S12. Glycoproteins identified in chia mucilage and the glycans attached to the proteins.

Protein Name	Prot Rank	Peptide	Glycans NHFAGNa	Mod. Type(s)	Observed m/z	z	Observed (M+H)	Mass error (ppm)	Starting position	Score	Delta	|Log Prob|	Unique peptides	Scan Time
Shispa_006g3054	13	R.LVVFDDPLTEGPN[+1170.417]LSSR.A	HexNAc(2)Hex(3)Fuc(1)Pent(1)	N[+1170]	1010.463	3	3029.373	0.5	75	707.3	707.3	12.91	11	81.8572
Shispa_006g3055	53	R.LAEGPN[+1170.417]LSSR.A	HexNAc(2)Hex(3)Fuc(1)Pent(1)	N[+1170]	1107.486	2	2213.965	-0.3	77	484.6	333.6	7.00	5	28.7229
Shispa_003g5435	22	R.EGPIIYLSN[+1170.417]TTHAPR.M	HexNAc(2)Hex(3)Fuc(1)Pent(1)	N[+1170]	947.102	3	2839.291	1.2	228	292.0	133.3	6.17	13	48.7226
Shispa_003g1874	34	R.TSSN[+1170.417]VTSFEGTNGQR.V	HexNAc(2)Hex(3)Fuc(1)Pent(1)	N[+1170]	1378.074	2	2755.140	-0.9	181	285.4	285.4	7.50	5	28.8115
Shispa_003g6730	29	K.SFAN[+203.079]LTK.I	HexNAc(1)	N[+203]	492.256	2	983.505	0.5	287	276.4	114.8	3.88	12	30.0366
Shispa_003g5250	102	R.GLLN[+1170.417]ETTIDK.A	HexNAc(2)Hex(3)Fuc(1)Pent(1)	N[+1170]	1137.511	2	2274.014	0.9	389	264.6	71.3	4.61	9	42.7815
Shispa_003g2699	164	K.ISTALSSN[+1170.417]K.T	HexNAc(2)Hex(3)Fuc(1)Pent(1)	N[+1170]	1045.964	2	2090.921	-0.8	794	239.5	161.3	4.99	5	17.6288
Shispa_006g3049	45	R.N[+1378.476]LSVVGGTGR.L	HexNAc(2)Hex(6)	N[+1378]	1169.505	2	2338.004	0.4	146	231.4	53.3	4.22	7	25.1641
Shispa_001g5228	56	K.N[+1170.417]TTDVGNAILGQEVTAR.I	HexNAc(2)Hex(3)Fuc(1)Pent(1)	N[+1170]	977.110	3	2929.316	0.0	174	186.8	186.8	5.15	4	57.5037
Shispa_001g2924	194	R.DALN[+568.212]ETGR.S	HexNAc(2)Hex(1)	N[+568]	722.318	2	1443.628	-3.5	206	83.2	64.2	0.95	2	30.6425
Shispa_002g2054	29	R.N[+1227.439]TTAGGWVAMDANTGK.I	HexNAc(3)Hex(3)Pent(1)	N[+1227]	941.063	3	2821.174	0.8	418	32.7	30.4	2.81	7	24.5135

Supplementary Table S13. Abundance of the proteins identified in *S. hispanica* mucilage[a].

Shispa Gene	Group	Avg Abundance	% abundance	% abundance accumulated
Shispa_002g1314.1	Seed storage prot	1138946371.4	**21.64**	21.64
Shispa_005g3469.1	Lipid metabolism	863903259.9	**16.42**	38.06
Shispa_001g4443.1	Seed storage prot	554973932.0	**10.55**	48.61
Shispa_004g3059.1	Seed storage prot	521282645.2	**9.91**	58.51
Shispa_004g3060.1	Seed storage prot	512336866.8	**9.74**	68.25
Shispa_001g4591.1	Seed storage prot	220522442.1	**4.19**	72.44
Shispa_006g1162.1	LEA	190320313.2	**3.62**	76.05
Shispa_002g1354.1	Seed storage prot	160511397.1	**3.05**	79.10
Shispa_003g3490.1	Seed storage prot	148591894.2	**2.82**	81.93
Shispa_002g1315.1	Seed storage prot	133789073.5	**2.54**	84.47
Shispa_003g2080.1	Seed storage prot	99883649.2	**1.90**	86.37
Shispa_006g3034.1	Seed maturation prot	95827543.3	**1.82**	88.19
Shispa_006g3054.1	Secondary Metabolism/Neolignans	84181838.8	**1.60**	89.79
Shispa_001g1166.1	Lipid metabolism	72994586.4	**1.39**	91.18
Shispa_004g3405.1	LEA	35115228.4	**0.67**	91.84
Shispa_001g3142.1	Lipid metabolism	30437191.8	**0.58**	92.42
Shispa_004g2159.1	Lipid metabolism	28011523.7	**0.53**	92.95
Shispa_003g1874.1	Lectin	22869125.7	**0.43**	93.39
Shispa_004g0434.1	Lipid metabolism	22235672.4	**0.42**	93.81
Shispa_003g5918.1	LEA	21169179.1	**0.40**	94.21
Shispa_003g6730.1	Cell wall	20920199.1	**0.40**	94.61
Shispa_001g1703.1	Proteolysis	18882035.7	**0.36**	94.97
Shispa_002g4364.1	Lipid metabolism	18040828.4	**0.34**	95.31
Shispa_005g1181.1	Not defined	16940725.9	**0.32**	95.63
Shispa_004g0690.1	LEA	15009217.1	**0.29**	95.92
Shispa_098g0002.1	Seed maturation prot	13238576.5	**0.25**	96.17
Shispa_003g5435.1	Seed storage prot	12441210.3	**0.24**	96.41
Shispa_005g3526.1	Seed storage prot	11404003.1	**0.22**	96.62
Shispa_001g5086.1	Secondary Metabolism/Neolignans	10951368.4	**0.21**	96.83
Shispa_002g0858.1	Carbohydrate metabolism	9798968.7	**0.19**	97.02
Shispa_001g0066.1	Lipid metabolism	8946290.0	**0.17**	97.19
Shispa_002g1357.1	LEA	8536266.7	**0.16**	97.35
Shispa_004g0670.1	Detoxification enzyme	8158219.5	**0.16**	97.51
Shispa_001g3236.1	Lipid metabolism	8043304.4	**0.15**	97.66
Shispa_003g6174.1	LEA	7948360.5	**0.15**	97.81
Shispa_013g0015.1	LEA	7579146.4	**0.14**	97.95
Shispa_001g4411.1	Seed storage prot	7309081.1	**0.14**	98.09
Shispa_003g0032.1	Lectin	4895391.3	0.09	98.19
Shispa_004g4150.1	Cell wall	4673406.9	0.09	98.27
Shispa_003g5163.1	Miscellaneous	4523625.3	0.09	98.36

Shispa_001g0500.1	Lipid metabolism	3998989.0	0.08	98.44
Shispa_001g3599.1	LEA	3951638.8	0.08	98.51
Shispa_002g2054.1	Not defined	3752560.5	0.07	98.58
Shispa_006g3049.1	Secondary Metabolism/Neolignans	3491756.6	0.07	98.65
Shispa_005g3551.1	Lipid metabolism	3372671.0	0.06	98.71
Shispa_005g4487.1	HSP/Chaperone	3140027.8	0.06	98.77
Shispa_002g3680.1	HSP/Chaperone	3082312.9	0.06	98.83
Shispa_003g6055.1	Seed storage prot	3080615.1	0.06	98.89
Shispa_002g3628.1	Detoxification enzyme	2746045.2	0.05	98.94
Shispa_001g5228.1	Lectin	2364627.9	0.04	98.99
Shispa_002g2912.1	Carbohydrate metabolism	2322081.6	0.04	99.03
Shispa_002g0414.1	Detoxification enzyme	2253448.2	0.04	99.07
Shispa_001g2823.1	HSP/Chaperone	2236666.6	0.04	99.12
Shispa_003g2568.1	Lipid metabolism	2225772.7	0.04	99.16
Shispa_003g2496.1	Detoxification enzyme	2215231.7	0.04	99.20
Shispa_006g3055.1	Secondary Metabolism/Neolignans	2135606.6	0.04	99.24
Shispa_003g4226.1	Carbohydrate metabolism	1962636.2	0.04	99.28
Shispa_004g2735.1	Proteolysis	1936887.6	0.04	99.32
Shispa_003g4214.1	Carbohydrate metabolism	1867007.4	0.04	99.35
Shispa_035g0015.1	Abiotic stress response	1836956.7	0.03	99.39
Shispa_002g0071.1	Seed storage prot	1833954.0	0.03	99.42
Shispa_001g5133.1	Not defined	1784721.5	0.03	99.45
Shispa_005g3993.1	Abiotic stress response	1692903.5	0.03	99.49
Shispa_003g6671.1	LEA	1638532.6	0.03	99.52
Shispa_005g3212.1	HSP/Chaperone	1589080.4	0.03	99.55
Shispa_003g4261.1	Seed storage prot	1584637.9	0.03	99.58
Shispa_004g4268.1	Not defined	1534631.0	0.03	99.61
Shispa_002g2707.1	Carbohydrate metabolism	1514136.1	0.03	99.64
Shispa_002g0035.1	Proteolysis	1510250.8	0.03	99.67
Shispa_002g4486.1	Proteolysis	1420237.6	0.03	99.69
Shispa_006g0641.1	Detoxification enzyme	1380826.9	0.03	99.72
Shispa_005g2441.1	Carbohydrate metabolism	1296383.2	0.02	99.74
Shispa_004g4416.1	Carbohydrate metabolism	1274224.4	0.02	99.77
Shispa_004g0747.1	HSP/Chaperone	1239288.5	0.02	99.79
Shispa_003g5250.1	Carbohydrate metabolism	1226011.2	0.02	99.81
Shispa_004g0402.1	Carbohydrate metabolism	1043353.0	0.02	99.83
Shispa_005g2087.1	Detoxification enzyme	882729.0	0.02	99.85
Shispa_005g5232.1	HSP/Chaperone	853467.9	0.02	99.87
Shispa_005g3878.1	Carbohydrate metabolism	760101.1	0.01	99.88
Shispa_005g7296.1	Proteolysis	651703.0	0.01	99.89
Shispa_004g2433.1	Detoxification enzyme	648861.4	0.01	99.91
Shispa_001g2798.1	HSP/Chaperone	597453.3	0.01	99.92
Shispa_006g2056.1	Carbohydrate metabolism	521091.8	0.01	99.93

Shispa_003g6688.1	Ribosomal	445418.2	0.01	99.94
Shispa_004g3318.1	Carbohydrate metabolism	444903.8	0.01	99.94
Shispa_003g2699.1	Carbohydrate metabolism	444767.6	0.01	99.95
Shispa_004g1651.1	Proteolysis	427304.0	0.01	99.96
Shispa_001g3634.1	Miscellaneous	376393.1	0.01	99.97
Shispa_001g2924.1	Carbohydrate metabolism	364932.5	0.01	99.97
Shispa_002g2928.1	HSP/Chaperone	364684.2	0.01	99.98
Shispa_003g0447.1	Miscellaneous	271016.3	0.01	99.99
Shispa_004g1835.1	HSP/Chaperone	261709.7	< 0.01	99.99
Shispa_005g4583.1	Miscellaneous	219115.0	< 0.01	100.00
Shispa_006g2139.1	Ribosomal	134596.9	< 0.01	100.00
Shispa_002g3117.1	Ribosomal	76837.6	< 0.01	100.00

Proteins with an abundance of at > 0.1% were defined as a high-confidence set of proteins present in *S. hispanica* seed mucilage (indicated in **bold**). [a]Average abundance of 3 technical replicates. Glycoproteins are indicated in blue.
