# Supplementary material for: Multi-omic analyses reveal the unique properties of chia (Salvia hispanica) seed metabolism
Source: Commun Biol. 2023 Aug 7;6:820. doi: 10.1038/s42003-023-05192-4 (PMC10406817; doi:10.1038/s42003-023-05192-4)
Supplement: Supplementary file 3 — Description of Additional Supplementary Files [file 42003_2023_5192_MOESM3_ESM.pdf]

Description of Additional Supplementary Files

File name: Supplementary Data S1

Description: aProteins identified in S. hispanica mucilage were functionally annotated using PANNZER, protein family assigned by InterPro, and best hit in Arabidopsis thaliana (blastp bitscore >= 80). Glycoproteins are indicated in blue. NA: Not assigned
